# Supplementary material for: Emergent climate protection strategies in German hospitals: A cluster analysis
Source: PLoS One. 2025 May 16;20(5):e0312661. doi: 10.1371/journal.pone.0312661 (PMC12083819; doi:10.1371/journal.pone.0312661)
Supplement: S1 Table — (DOCX) [file pone.0312661.s001.docx]

**Supporting Information 1**

**Table 1. Socio-demographic characteristics of the respondents (n=205).**

| **Characteristics** | **Sample (%)** |
| --- | --- |
| **Age** | |
| Under 30 | 7.32 % |
| 30 – 39 | 19.02 % |
| 40 – 49 | 23.41 % |
| 50 – 59 | 35.12 % |
| Over 60 | 15.12 % |
| **Gender** | |
| Male | 81.46 % |
| Female | 18.05 % |
| Diverse | 0.49 % |
| **Administrative leadership position in multiple hospitals** | |
| Yes | 36.10 % |
| No | 63.90 % |
| **Work experience as a hospital administrative leader** | |
| Less than 5 years | 20.00 % |
| 5 – 9 years | 17.56 % |
| 10 – 14 years | 18.05 % |
| 15 – 20 years | 18.05 % |
| More than 20 years | 26.34 % |

**Table 2: Hospital characteristics (n=205).**

| **Characteristics** | **Sample (%)** |
| --- | --- |
| **Hospital beds** | |
| Less than 50 | 2.44 % |
| 50 – 149 | 17.56 % |
| 150 – 299 | 20.00 % |
| 300 – 499 | 25.85 % |
| 500 – 799 | 19.02 % |
| More than 800 | 15.12 % |
| **Level of care** | |
| Specialist care | 18.05 % |
| Primary and standard care | 39.02 % |
| Intermediate care | 27.32 % |
| Maximum care | 8.78 % |
| University clinic | 6.83 % |
| **Hospital ownership** | |
| Public | 38.54 % |
| Private non-profit | 33.17 % |
| Private for-profit | 28.29 % |
| **Hospital structure** | |
| Single hospital | 40.00 % |
| Hospital alliance | 24.88 % |
| Hospital group | 35.12 % |
